# Low Protein Diet Improves Meat Quality and Modulates the Composition of Gut Microbiota in Finishing Pigs

**DOI:** 10.3389/fvets.2022.843957

**Published:** 2022-05-17

**Authors:** Cui Zhu, Jingsen Yang, Qiwen Wu, Jingping Chen, Xuefen Yang, Li Wang, Zongyong Jiang

**Affiliations:** ^1^Institute of Animal Science, Guangdong Academy of Agricultural Sciences, State Key Laboratory of Livestock and Poultry Breeding, Ministry of Agriculture Key Laboratory of Animal Nutrition and Feed Science in South China, Guangdong Provincial Key Laboratory of Animal Breeding and Nutrition, Maoming Branch, Guangdong Laboratory for Lingnan Modern Agriculture, Guangzhou, China; ^2^School of Life Science and Engineering, Foshan University, Foshan, China

**Keywords:** low protein diet, finishing pig, meat quality, gut microbiota, antioxidant capacity

## Abstract

This study investigated the effect of a low protein (LP) diet on growth performance, nitrogen emission, carcass traits, meat quality, and gut microbiota in finishing pigs. Fifty-four barrows (Duroc × Landrace × Yorkshire) were randomly assigned to three treatments with six replicates (pens) of three pigs each. The pigs were fed with either high protein (HP, 16% CP), medium protein (MP, 12% CP), and LP diets (10% CP), respectively. The LP diets did not influence the growth performance, but significantly decreased the plasma urea nitrogen contents and fecal nitrogen emission (*P* < 0.05). The LP diet significantly decreased the plasma contents of malondialdehyde (MDA) and increased the plasma glutathione (GSH) contents (*P* < 0.05). The LP diets significantly increased the backfat thickness at the first and last ribs, L^*^ (lightness) value of meat color, and muscle fiber density in the *longissimus dorsi* (*P* < 0.05). The messenger RNA (mRNA) expression of fatty acid synthetase (*FAS*), peroxisome proliferator-activated receptor-gamma (*PPAR*γ), *leptin*, and acetyl-CoA carboxylase (*ACC*) was significantly downregulated, while that of carnitine palmitoyltransferase 1 (*CPT*1) and myosin heavy chain (*MYHC*) *IIx* in the *longissimus Dorsi muscle* was significantly upregulated by LP diets (*P* < 0.05). The 16S sequencing analysis showed that the abundance of unidentified Bacteria at the phylum level, and *Halanaerobium* and *Butyricicoccus*at at the genus level in the colonic digesta were significantly decreased by LP diet (*P* < 0.05). The LP diet significantly decreased the observed species of α-diversity in both ileal and colonic microbiota (*P* < 0.05). Spearman correlation analysis identified a significant positive correlation between the abundance of the ileal genera *Streptococcus* and L^*^ value at 24 and 48 h, and a significant negative correlation between unidentified_*Ruminococcasceae* in both ileum and colon with L^*^ value at 24 h (*P* < 0.05). Collectively, the LP diet supplemented with lysine, methionine, threonine, and tryptophan could reduce the fecal nitrogen emission without affecting growth performance and improve meat quality by regulating the antioxidant capacity and gene expression involved in fat metabolism as well as modulating the gut microbiota composition in finishing pigs.

## Introduction

Dietary protein functions as the fundamental source of amino acids (AAs) for livestock. However, the high inclusion of dietary protein and the imbalance of AA composition in animal husbandry could result in inefficient utilization of protein resources and severe nitrogen pollution (1). Notably, the shortage of high-quality protein sources and nitrogen excretion environmental pollution represent a worldwide problem, especially in China (2). Thus, reducing dietary crude protein (CP) content can effectively alleviate the pressure on protein ingredient supply and reduce the nitrogen excretion in feces and urine, which contributes to healthy and sustainable animal husbandry in an economical and environment-friendly way (1, 2). The low protein (LP) diet has been widely shown to reduce the incidence of post-weaning diarrhea by maintaining gut health through improvement of intestinal development and barrier function, and modulation of gut microbial community and metabolites in weaned piglets (3, 4). Moreover, a moderate reduction of dietary protein concentration (13% CP) could improve the bacterial community structure in both the ileum and colon of adult pigs (2). Previous studies have shown that an LP diet with supplemental AAs could improve the meat quality and carcass compositions of pigs (5–7). However, whether the improvement of carcass traits and meat quality by LP diet was mediated through the modulation of gut microbiota in finishing pigs remained largely unclear. Therefore, this study was carried out to investigate the effects of LP diets supplemented with lysine, methionine, threonine, and tryptophan on the growth performance, nitrogen emission, carcass characteristics, meat quality, antioxidant capacity, and gut microbiota in finishing pigs.

## Materials and Methods

### Animals and Experimental Diets

A total of 54 crossbred barrows (Duroc × Landrace × Yorkshire) with similar initial body weight (BW, 100.38 ± 0.97 kg) were randomly assigned to three dietary treatments. The pigs were fed with a high protein (**HP**) diet (16% CP), a medium protein (**MP**) diet (12% CP), or a low protein (**LP**) diet (10% CP). Each treatment had six replicates (pens) of three pigs each. The diets (Table 1) were formulated based on the nutritional requirements of the National Research Council (**NRC**) recommended for finishing pigs (8). The four supplemental AAs (lysine, methionine, threonine, and tryptophan) were added to meet their requirements and maintain AA balance among groups. The levels of dietary energy and standardized ileal digestibility (**SID**) value of AAs were maintained consistently among treatments. The experiment lasted until the pigs reached around 130 kg. Pigs had free access to feed and water throughout the whole experiment.

**Table 1:** Ingredient and nutrient levels of the diets for finishing pigs.

**Ingredients**	**Dietary treatment groups** ^a^		
	**HP**	**MP**	**LP**
Corn	75.34	85.60	90.68
Soybean meal	21.76	9.73	3.70
Soybean oil	0.00	1.00	1.53
Lysine	0.10	0.46	0.64
Methionine	0.00	0.05	0.07
Tryptophan	0.00	0.06	0.10
Threonine	0.00	0.15	0.23
Limestone	0.65	0.65	0.65
CaHPO_4_	0.85	1.00	1.10
NaCl	0.30	0.30	0.30
Premix^b^	1.00	1.00	1.00
Total	100.00	100.00	100.00
**Nutrient levels,%**			
DE, kcal/kg	3447	3448	3449
ME, kcal/kg	3339	3364	3378
NE, kcal/kg	2448	2448	2448
CP	16.00	12.01	10.01
Ca	0.51	0.51	0.52
Total phosphorus	0.49	0.47	0.46
Available phosphorus	0.27	0.28	0.29
SID Lysine	0.83	0.83	0.83
SID Methionine	0.25	0.25	0.25
SID Methionine + cystine	0.54	0.47	0.44
SID Threonine	0.53	0.53	0.53
SID Tryptophan	0.17	0.17	0.17

a *Dietary treatment: HP, high protein (16% crude protein); MP, medium protein (12% crude protein); LP, low protein (10% crude protein)*.

b *Supplied per kg of diet: vitamin A, 1,300 IU; vitamin D_3_, 150 IU; vitamin E, 11 mg; vitamin K_3_, 0.50 mg; vitamin K, 0.50 mg; biotin, 0.05 mg; choline, 0.30 mg; folic acid, 30 mg; calcium pantothenate, 7 mg; vitamin B_1_, 1 mg; vitamin B_2_, 2 mg; vitamin B_6_, 5 mg; vitamin B_12_, 0.05 mg; Cu, 3 mg; Fe, 40 mg; Zn, 50 mg; Mn, 2 mg; I, 0.14 mg; Se, 0.15 mg*.

### Growth Performance

Each pig was weighed individually at d 1, 14, and 28 of the experiment. The growth performance including average daily gain (ADG), average daily feed intake (ADFI), and feed to gain ratio (F/G) during the experiment was calculated accordingly on a pen basis.

### Sample Collections

At the end of the experiment, one pig with average weight was selected from each replicate (pen) for blood and sample collections. Briefly, the pigs were fed with the last meal at 18:00 h, and the blood samples were collected from the inferior auricular vein of selected pigs from each replicate at 08:00 h the next morning. The plasma samples were centrifuged at 1,000 *g* and 4°C for 10 min and stored at −80°C until analysis for biochemical parameters. After blood collection, the pigs were rinsed with cool water, electrically stunned, and subjected to slaughter. Samples of the *longissimus dorsi*, at the 10th rib of the right carcass, were collected for RNA extraction and further gene expression determinations. The intestinal digest the of ileum and colon were also collected for microbial analyses. The samples were immediately frozen in liquid nitrogen followed by storage at −80°C until analysis.

### Analyses of Plasma Biochemical Parameters and Hormone Levels

The plasma biochemical parameters including the lactate, glucose, albumin, triglyceride, cholesterol, total protein, and urea nitrogen (**PUN**) concentrations, were determined using the recommended dilutions of samples with standard commercial kits following the manufacturer's instructions using an Architect C8000 Automatic Biochemical Analyzer (Abbott, Inc., Chicago, IL, USA). The plasma hormones corticotropin-releasing hormone (**CRH**, #H288), adreno-cortico-tropic-hormone (**ACTH**, #H097-1-2), cortisol (#H094), and insulin (#H203-1-2) levels were determined according to the instructions of the commercial kits (Nanjing Jiancheng Bioengineering Institute, Nanjing, China). The standard curves were created accordingly as the manufacturer recommended.

### Analyses of Plasma Antioxidant Indexes

The plasma antioxidant indexes include the malondialdehyde (MDA, #A003-3-1), glutathione (GSH, #A006-2-1), glutathione oxidized (GSSG, #A061-1-2), GSH/GSSG, and reactive oxygen species (ROS, #E004-1-1) levels, and the activities of catalase (CAT, #A007-2-1), total superoxide dismutase (T-SOD, #A001-1-2), and glutathione peroxidase (GSH-Px, #A005-1-2) were measured with the corresponding commercial kits (Nanjing Jiancheng Bioengineering Institute, Nanjing, China) following the manufacturer's instructions. The results of plasma antioxidant indexes were calculated with their corresponding standard curves accordingly.

### Carcass Traits

Immediately after slaughter, the individual hot carcass weight was recorded, and the dressing percentage was calculated accordingly. Abdominal fat was harvested and individually weighed to calculate its percentage. The left carcass was used to determine the carcass traits. The loin area of the *longissimus dorsi* was determined at the tenth-eleventh rib interface with the vitriol paper and measured with the compensating planimeter (Model Q811; Xinanjiang Science Instrument Factory, Zhejiang, China). The thickness of the back fat at the first, tenth, last, and last lumbar vertebra was measured with the vernier caliper (RH OMBI 5-32294, Guangzhou, China). Daily gain of fat-free lean meat was calculated using the hot carcass weight, backfat thickness, and loin area values according to the equation recommended by National Pork Producers Council (**NPPC**) (9). Moreover, the protein and fat retention were calculated based on the equation recommended by NRC (8).

### Meat Quality Measurements

After determining the loin area, the *longissimus Dorsi* muscle samples at the tenth-fourteenth rib interface of the left carcass were collected and separated into several chops for meat quality measurements. Briefly, a chop (25-mm thick slice) from *longissimus dorsi* between the 10th and 11th ribs was used to measure meat color at 45 min, 24 h, and 48 h after slaughter using a Minolta CR-400 Chroma Meter (Konica Minolta Sensing Ins., Osaka, Japan). The values of L^*^ (lightness), a^*^ (redness), and b^*^ (yellowness) of each sample were recorded accordingly. After meat color determination, the samples were used to measure pH values at 45 min, 24 h, and 48 h postmortem using a pH meter (HI 8242C, Beijing Hanna Instruments Science and Technology, Beijing, China) equipped with an insertion glass electrode.

Another chop (25 mm thick slice) was cut from each pig between the 11th and 12th ribs for determining the marbling score according to the marbling testing standard recommended by NPPC (9). Moreover, ~30 g of fresh muscle sample between the 12th and 13th ribs was used for analyzing the intramuscular fat (IMF) content using the Soxhlet extraction method. Briefly, each sample was processed in a meat grinder (Philips, Eindhoven, The Netherlands) and lyophilized using a freeze-drying system (Marin Chris, Osterode, Germany) after removing the visible fascia. The sample was then grounded into powder (3 g) and extracted with petroleum ether and analyzed for ether extracts (EE) using the Soxtec 2055 fat extraction system (Foss Tecator AB, Hilleroed, Denmark) by the methods of the Association of Official Analytical Chemists (AOAC) (10).

Drip loss was determined by cutting three cylindrical muscle cubes (about 30 g) from another muscle chop (25-mm thick slice) of *longissimus dorsi* between the 13th and 14th ribs, weighed individually, and then, wrapped in airtight plastic bags, and stored at 4°C for 24 h and 48 h. The muscle samples were weighed individually at 24 h and reweighed at 48 h after removing surface moisture. Drip loss was estimated by calculating the difference between the initial and the final weights of each muscle sample.

After determining the marbling score, the sample of *longissimus dorsi* between the 11th and 12th ribs was used for assessing the muscle fiber density according to the methods described previously (11). Briefly, *longissimus dorsi* muscle samples (about 1 cm^3^) were fixed in 10% formalin for 24 h, dehydrated with graded alcohol, and then, mounted with paraffin wax followed by staining with hematoxylin and eosin (HE). Each section was photographed with five views under a light microscope (Model BH-2; Olympus, Tokyo, Japan) equipped with the image analysis software (Model 3.1; Motic, Xiamen, Fujian, China), and muscle fiber density was calculated accordingly.

### Determinations of Dietary CP, Nitrogen, and Phosphorus in the Feces

Nitrogen in the feces and dietary CP levels were determined using an N analyzer (Kjeltec 8400, FOSS Analytical AB, Sweden) following the instructions recommended by the National Standard of the People's Republic of China (GB/T 6432-2018). The fecal phosphorus concentration was analyzed according to the instructions of the National Standard of the People's Republic of China (GB/T 6437-2018).

### Quantitative Real-Time PCR (qPCR) Analysis

Total RNA was extracted from fresh *longissimus dorsi* tissues using TRIzol™ reagent (Invitrogen, CA, USA) according to the manufacturers' instructions. The concentration and purity of extracted RNA were measured at 260/280 nm using a NanoDrop-ND1000 spectrophotometer (Thermo Scientific, USA). Total RNA (1 μg) was used to synthesize the cDNA using a PrimeScript™ II First Strand cDNA Synthesis Kit (Takara, Tokyo, Japan) according to the manufacturer's instructions. Real-time PCR was performed in a final volume of 20 μL using an ABI 7500 Mastercycler (Applied Biosystems, CT, USA) with TaqMan™ PCR Mix (TaKaRa, Dalian, China) according to the manufacturers' instructions. Primers (Table 2) were designed and synthesized by Shanghai Boya (Shanghai, China). The real-time quantitative PCR cycling conditions used were as follows: 95°C for 30 s, followed by 40 cycles at 95°C for 5 s and 60°C for 20 s, and the last cycle at 72°C for 35 s. The relative mRNA expression of targeted genes was expressed as relative to that of the housekeeping gene (β*-actin*) using the 2–^ΔΔ*Ct*^ method.

**Table 2:** Sequences and accession number of primers for targeted genes.

**Primer**	**Sequence (5' to 3')**	**Accession no**.
*FAS*	F: AGCCTAACTCCTCGCTGCAAT R: TCCTTGGAACCGTCTGTGTTC	AF296508.1
*LPL*	F: AACTTGTGGCTGCCCTAT R:GACCCTCTGGTGAATGTG	AY559454.1
*HSL*	F: GCTCCCATCGTCAAGAATC R:TAAAGCGAATGCGGTCC	S80110.1
*FABP3*	F: CGCCTGTTCTGTCGTCTC R: TCTCATCAAACTCCACTCCC	NM_001099931.1
*PPARγ*	F: GATTTCTCCAGCATTTCCA R: GCTCTTCGTGAGGTTTGTT	AY887104.1
*Leptin*	F: GGCTCCCGAGGTGCTGTT R: GAACCTGGCCCTTCGAGATC	GQ240885.1
*CPT1*	F: ATGGTGGGCGACTAACT R: TGCCTGCTGTCTGTGAG	AF284832.1
*ACC*	F: ATGTTTCGGCAGTCCCTGAT R: TGTGGACCAGCTGACCTTGA	AF175308.1
*MyHC I*	F: AAGGGCTTGAACGAGGAGTAGA R: TTATTCTGCTTCCTCCAAAGGG	L10129
*MyHC IIa*	F: GCTGAGCGAGCTGAAATCC R: ACTGAGACACCAGAGCTTCT	U11772
*MyHC IIx*	F: AGAAGATCAACTGAGTGAACT R: AGAGCTGAGAAACTAACGTG	U90720
*Myostatin*	F: AATGAGAACAGCGAGCAA R: TTCCGTCGTAGCGTGATA	AH010972.2
*β-actin*	F: CATCGTCCACCGCAAAT R: TGTCACCTTCACCGTTCC	NC_010445

*LPL, Lipoprteinlipase; FAS, fatty acid synthetase; HSL, hormone-sensitive lipase; FABP3, fatty acid binding protein 3; PPARγ, peroxisome proliferator activated receptor gamma; CPT1, carnitine palmitoyl transferase 1; ACC, acetyl-CoA carboxylase; MYHC, myosin heavy chain*.

### Microbial DNA Extraction, 16S rRNA Amplification, and Analyses of Illumina Sequencing Data

The microbial genomic DNA was extracted from the contents of the ileum and colon using the QIAamp DNA Stool Mini Kit (Qiagen, Germany) according to the manufacturer's instructions. The DNA quantity and quality of DNA were assessed by a Nanodrop ND-1000 spectrophotometer (Thermo Scientific, USA) and checked by 1% agarose gel electrophoresis. The V3-V4 hypervariable region of the bacterial 16S rRNA gene was amplified using the specific primers with barcodes (341 F: 5'-CCTAYGGGRBGCASCAG-3'; 806 R: 5'-GGACTACNNGGGTATCTAAT-3'). Illumina sequencing of the libraries was performed using the Ion Plus Fragment Library Kit (48 rxns, Thermo Fisher Scientific Inc., MA, USA) following the manufacturer's instructions on HiSeq 2500 PE250 platform (Illumina, USA) according to the standard protocols by Novogene Technology Co., Ltd. (Beijing, China).

Bioinformatic analysis of 16S rRNA sequencing data was conducted using quantitative insights into microbial ecology *via* the QIIME software package (12). After filtering the raw data for clean sequence, the effective sequence reads, with 97% sequence similarity, were picked to build distinct operational taxonomic units (**OTU**). Each representative sequence was annotated with its taxonomic information based on Mothuralgorithm using the Silva 132 database (http://www.arb-silva.de/). The Venn diagram with shared and unique OTUs was used to identify the similarities and differences among treatments. The core-metrics command of the diversity plugin was used to calculate bacterial alpha diversity and beta diversity, and the effect on alpha and beta diversity was assessed using some indices. The alpha-diversity within groups included Observed_species, Shannon index, Simpson index, Chao1 richness, abundance-based coverage estimators (ACE), Goods_coverage, and phylogenetic diversity (PD) of the whole tree (PD_whole_tree) using QIIME software (version 1.9.1). The β-diversity index, principal coordinate analysis (PCoA plots, Bray-Curtis), and Unweighted Pair-group Method with Arithmetic Means (UPGMA) clustering tree were used to indicate the bacterial beta diversity among groups by QIIME software. The difference in the relative abundances of gut microbiota among treatments was compared using the linear discriminant analysis effect size (LEfSe) by LEfSe software (version 1.0), analysis of similarities (ANOSIM), and *t*-test methods using R software (version 2.15.3).

### Statistical Analyses

Data were analyzed by the one-way ANOVA using IBM SPSS Statistics software (version 22.0, Chicago, USA). The difference in means among groups was compared by Duncan's multiple comparisons. The results are expressed as means ± standard error (SE). Correlations between the gut microbiota and meat quality were analyzed by Pearson's correlation. *P* < 0.05 were considered statistically significant and *P* < 0.10 indicated a trend.

## Results

### Growth Performance and Emissions of Fecal Nitrogen and Phosphorus

**Figure 1:**
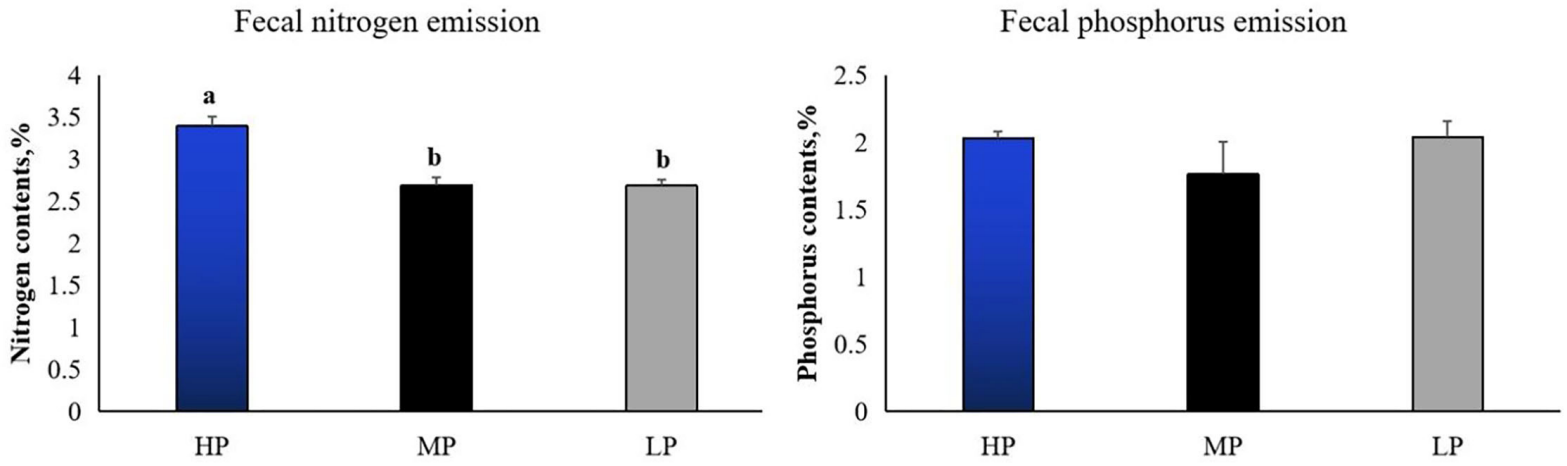
Effect of low-protein diet on the fecal nitrogen and phosphorus emissions in finishing pigs. HP, high protein (16% crude protein); MP, medium protein (12% crude protein); LP, low protein (10% crude protein). ^a−b^Column with different numbers indicate that there is a significant difference among different treatments (*P* < 0.05).

Growth performance is shown in Table 3. There were no significant differences in body weight (BW) at d 1, 14, and 28, as well as the average daily gain (ADG), average daily feed intake (ADFI), and feed to gain ratio (F/G) at either d 1–14, d 14–28, or d 1–28 in finishing pigs among the MP, LP and HP treatments (*P* > 0.5). However, the fecal nitrogen emission was significantly decreased by the LP diet compared to the HP diet (*P* < 0.05) (Figure 1) although the fecal phosphorus emission was not affected by dietary treatments (*P* > 0.5).

**Table 3:** Effect of low protein diet on the growth performance in finishing pig.

**Item**	**HP**	**MP**	**LP**	***P*** **-value**
**BW, kg**				
d1	100.53 ± 1.74	100.42 ± 1.84	100.19 ± 1.77	0.991
d14	116.70 ± 1.47	111.83 ± 0.95	113.47 ± 1.51	0.080
d28	128.33 ± 1.88	128.30 ± 2.83	127.90 ± 1.39	0.461
**ADG, kg/d**				
d1–14	1.09 ± 0.06	0.91 ± 0.08	0.95 ± 0.06	0.209
d14–28	0.83 ± 0.12	0.93 ± 0.11	0.96 ± 0.08	0.645
d1–28	0.96 ± 0.07	0.92 ± 0.03	0.95 ± 0.06	0.879
**ADFI, kg/d**				
d1–14	3.22 ± 0.04	3.39 ± 0.05	3.34 ± 0.05	0.103
d14–28	2.62 ± 0.24	3.00 ± 0.19	2.64 ± 0.15	0.333
d1–28	2.93 ± 0.14	3.20 ± 0.07	2.98 ± 0.07	0.108
**F/G**				
d1–14	3.02 ± 0.15	3.81 ± 0.33	3.57 ± 0.16	0.074
d14–28	3.31 ± 0.35	3.39 ± 0.34	2.87 ± 0.32	0.502
d1–28	3.12 ± 0.19	3.50 ± 0.17	3.18 ± 0.21	0.362

*HP, high protein (16% crude protein); MP, medium protein (12% crude protein); LP, low protein (10% crude protein); BW, body weight; ADG, average daily gain; ADFI, average daily feed intake; F/G, feed to gain ratio*.

### Carcass Traits

As shown in Table 4, the LP diet significantly increased the backfat thickness of the first rib and the last rib compared with other treatment groups (*P* < 0.05). However, the LP diet did not significantly affect the carcass weight, dressing percentage, loin area, abdominal fat, a daily gain of non-fat lean meat, protein retention, fat retention, and backfat thickness of the 10th rib and the last lumbar in finishing pigs (*P* > 0.5).

**Table 4:** Effect of low protein diet on the carcass traits in finishing pig.

**Item**	**HP**	**MP**	**LP**	***P*** **-value**
Carcass weight, kg	93.88 ± 1.01	93.48 ± 1.74	96.53 ± 0.88	0.215
Dressing percentage, %	73.67 ± 0.29	73.70 ± 0.46	74.50 ± 0.55	0.356
Loin area, cm^2^	62.16 ± 2.97	57.59 ± 2.27	60.83 ± 1.81	0.390
Abdominal fat, %	1.69 ± 0.07	1.49 ± 0.10	1.72 ± 0.08	0.168
Daily gain of non-fat lean meat, g/d	290.81 ± 78.83	269.88 ± 33.44	287.61 ± 69.27	0.858
Protein retention, g/d	122.96 ± 1.18	122.36 ± 1.53	122.92 ± 1.33	0.582
Fat retention, g/d	358.89 ± 1.82	356.42 ± 2.47	355.62 ± 2.20	0.553
**Backfat thickness (cm)**				
First rib	4.57 ± 0.18^b^	5.25 ± 0.16^a^	5.47 ± 0.27^a^	0.022
Tenth rib	3.68 ± 0.14	3.55 ± 0.05	4.08 ± 0.18	0.055
Last rib	3.12 ± 0.18^b^	3.60 ± 0.19^ab^	3.76 ± 0.15^a^	0.049
Last lumbar	3.33 ± 0.10	3.13 ± 0.14	3.28 ± 0.14	0.546

*HP, high protein (16% crude protein); MP, medium protein (12% crude protein); LP, low protein (10% crude protein)*.

a, b *Within a row, means (n = 6) with a different superscript are significantly different at P < 0.05*.

### Meat Quality

The LP diet significantly increased the value of L^*^ at 24 h and the muscle fibers density in finishing pigs when compared to those fed HP diets (*P* < 0.05) (Table 5). In addition, compared with other groups, the value of L^*^ at 48 h in finishing pigs fed with the LP diets was significantly increased (*P* < 0.05). Furthermore, the muscle fibers density was significantly increased by the LP diet when compared to the HP diet (*P* < 0.05). However, there were no significant effects on IMF, marbling score, redness (a^*^) and yellowness (b^*^), pH value, and drip loss of finishing pigs among the three groups (*P* > 0.5).

**Table 5:** Effect of low protein diet on the meat quality in finishing pig.

**Item**	**HP**	**MP**	**LP**	***P*** **-value**
IMF, %	3.02 ± 0.27	3.05 ± 0.15	2.71 ± 0.32	0.596
Marbling score	2.40 ± 0.14	2.72 ± 0.13	2.86 ± 0.09	0.071
L^*^				
45 min	42.03 ± 0.67	41.60 ± 0.08	43.17 ± 0.75	0.203
24 h	52.65 ± 0.49^b^	54.02 ± 0.08^ab^	55.17 ± 0.61^a^	0.029
48 h	53.93 ± 0.60^b^	54.25 ± 0.48^b^	56.73 ± 0.49^a^	0.016
a^*^				
45 min	20.71 ± 0.60	18.93 ± 0.58	20.18 ± 0.88	0.237
24 h	18.18 ± 1.61	18.02 ± 1.29	17.99 ± 1.67	0.996
48 h	19.36 ± 1.23	19.54 ± 0.61	19.39 ± 0.92	0.990
b^*^				
45 min	2.18 ± 0.18	2.35 ± 0.24	2.62 ± 0.04	0.478
24 h	3.26 ± 0.06	3.20 ± 0.19	3.35 ± 0.29	0.900
48 h	2.70 ± 0.13	3.24 ± 0.05	3.32 ± 0.23	0.430
**Drip loss**				
24 h	0.20 ± 0.00	0.21 ± 0.01	0.22 ± 0.01	0.446
48 h	0.26 ± 0.01	0.27 ± 0.01	0.25 ± 0.01	0.289
**pH**				
45 min	6.12 ± 0.14	6.15 ± 0.05	6.31 ± 0.13	0.486
24 h	5.40 ± 0.05	5.44 ± 0.03	5.38 ± 0.04	0.582
48 h	5.41 ± 0.03	5.43 ± 0.01	5.40 ± 0.03	0.776
Muscle fibers density (number/mm^2^)	281.01 ± 8.53^b^	317.12 ± 10.89^ab^	337.95 ± 11.64^a^	0.032

*HP, high protein (16% crude protein); MP, medium protein (12% crude protein); LP, low protein (10% crude protein); IMF, intramuscular fat*.

a, b *Within a row, means (n = 6) with a different superscript are significantly different at P < 0.05*.

### Plasma Biochemical Indexes and Hormone Levels

In comparison with the MP diet, the LP diet significantly reduced the plasma total protein, urea nitrogen, triglyceride contents, and plasma cortisol level in finishing pigs (*P* < 0.05) (Table 6). Compared with other treatment groups, the LP diet significantly reduced the plasma ACTH level in finishing pigs (*P* < 0.05). However, there were no significant effects of LP diets on the plasma levels of lactate, glucose, albumin, cholesterol, and insulin in finishing pigs (*P* > 0.5).

**Table 6:** Effect of low protein diet on plasma biochemical index and hormone levels in finishing pig.

**Item**	**HP**	**MP**	**LP**	***P*** **-value**
Lactate, mmol/L	2.54 ± 0.24	2.73 ± 0.22	3.03 ± 0.17	0.348
Glucose, mmol/L	4.49 ± 0.17	4.56 ± 0.16	4.31 ± 0.15	0.555
Albumin, g/L	43.68 ± 1.70	39.70 ± 1.30	41.45 ± 0.76	0.127
Triglyceride, mmol/L	0.21 ± 0.03^b^	0.27 ± 0.04^a^	0.16 ± 0.01^c^	0.003
Cholesterol, mmol/L	3.12 ± 0.14	3.87 ± 0.21	3.47 ± 0.26	0.090
Total protein, μg/mL	65,440.74 ± 1,097.75^ab^	68,104.37 ± 877.31^a^	62,384.13 ± 1,161.88^b^	0.013
Urea nitrogen, ng/mL	3.06 ± 0.09^a^	2.68 ± 0.27^ab^	2.33 ± 0.08^b^	0.020
CRH, ng/L	259.98 ± 15.63^b^	323.32 ± 29.75^a^	318.90 ± 10.87^a^	0.039
ACTH, ng/mL	13.26 ± 1.15^b^	15.86 ± 1.87^b^	21.53 ± 2.50^a^	0.002
Cortisol, ng/mL	100.04 ± 14.59^a^	108.30 ± 9.40^a^	55.21 ± 13.11^b^	0.036
Insulin, ng/mL	7.70 ± 0.76	8.90 ± 0.49	8.34 ± 0.93	0.534

a, b *Within a row, means (n = 6) with a different superscript are significantly different at P < 0.05*.

*HP, high protein (16% crude protein); MP, medium protein (12% crude protein); LP, low protein (10% crude protein); CRH, corticotropin releasing hormone; ACTH, adreno-cortico-tropic-hormone*.

### Plasma Antioxidant Capacity

As shown in Table 7, the LP diet significantly decreased the contents of MDA and GSSG in plasma, and significantly increased the plasma content of GSH and the ratio of GSH/GSSG (*P* < 0.05). However, there were no significant differences in the plasma contents of CAT, T-SOD, GSH-Px, and ROS among the treatments (*P* > 0.05).

**Table 7:** Effect of low protein diet on the p antioxidant capacity in finishing pig.

**Item**	**HP**	**MP**	**LP**	***P*** **-value**
MDA, nmol/mL	1.25 ± 0.15^b^	1.59 ± 0.05^a^	1.02 ± 0.07^b^	0.020
CAT, U/mL	2.17 ± 0.14	2.12 ± 0.21	1.99 ± 0.28	0.780
T-SOD, U/mL	130.88 ± 6.11	144.55 ± 3.55	143.32 ± 4.18	0.133
GSH-Px, U/mL	638.19 ± 20.91	565.94 ± 9.27	620.59 ± 22.13	0.094
GSH, μmol/L	3.15 ± 0.31^b^	2.81 ± 0.39^b^	5.63 ± 0.33^a^	0.038
GSSG, μmol/L	11.29 ± 0.60^a^	7.36 ± 0.55^b^	8.18 ± 0.69^b^	0.003
GSH/GSSG	0.32 ± 0.06^b^	0.34 ± 0.02^b^	0.70 ± 0.04^a^	0.003
ROS, U/mL	26.56 ± 1.05	26.32 ± 0.91	28.55 ± 0.93	0.278

a, b *Within a row, means (n = 6) with a different superscript are significantly different at P < 0.05*.

*HP, high protein (16% crude protein); MP, medium protein (12% crude protein); LP, low protein (10% crude protein); MDA, Malondialdehyde; CAT, Catalase; T-SOD, Total superoxide dismutase; GSH-Px, Glutathione peroxidase; GSH, Glutathione; GSSG, Glutathione Oxidized; ROS, Reactive Oxygen Species*.

### The mRNA Expression of Genes Related to Fat Metabolism and Muscle Fiber Types by qPCR

**Figure 2:**
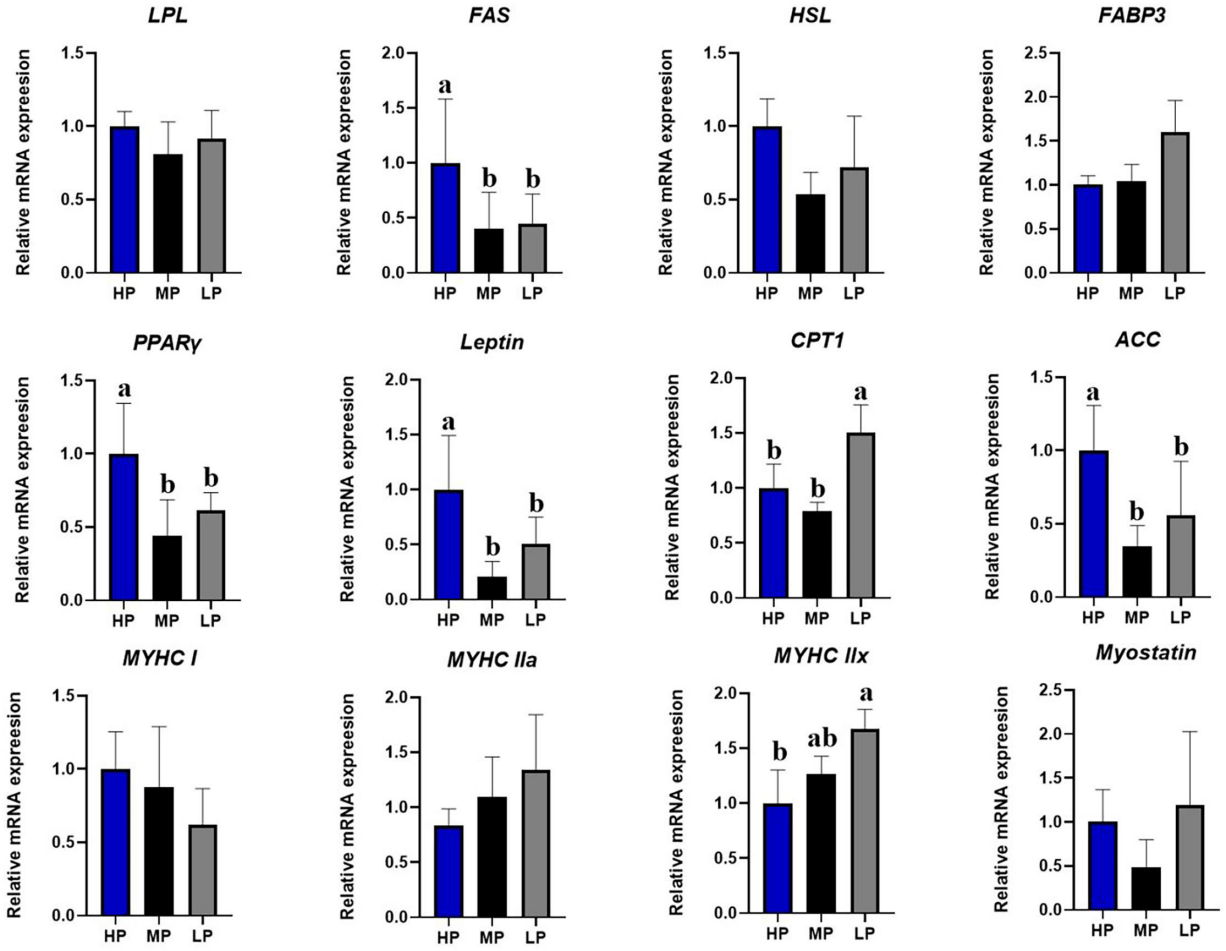
Effect of low-protein diet on the relative mRNA expression levels of muscle genes in finishing pigs. LPL, Lipoprteinlipase; FAS, fatty acid synthetase; HSL, hormone-sensitive lipase; FABP3, fatty acid binding protein 3; PPARγ, peroxisome proliferator activated receptor gamma; CPT1, carnitine palmitoyl transferase 1; ACC, acetyl-CoA carboxylase; MYHC, myosin heavy chain; HP, high protein (16% crude protein); MP, medium protein (12% crude protein); LP, low protein (10% crude protein). ^a−c^Column with different numbers indicate that there is a significant difference among different treatments (*P* < 0.05).

As shown in Figure 2, compared with the HP diet, the LP diet significantly reduced the mRNA expression of fatty acid synthetase (*FAS*), peroxisome proliferator-activated receptor-gamma (*PPAR*γ), *leptin*, and acetyl-CoA carboxylase (*ACC*) mRNA in the *longissimus dorsi* muscle of finishing pigs (*P* < 0.05) (Figure 2). Moreover, the LP diet significantly increased the mRNA expression of carnitine palmitoyltransferase 1 (*CPT*1) and myosin heavy chain (*MYHC*) *IIx* in the *longissimus dorsi* muscle of finishing pigs (*P* < 0.05). However, no significant effects were observed in the mRNA expression of Lipoprotein lipase (*LPL*), hormone-sensitive lipase (*HSL*), fatty acid-binding protein 3 (*FABP3*), *MYHC I*, MYHC *II*a, and *myostatin* in the *longissimus dorsi* muscle of finishing pigs among different groups (*P* > 0.5).

### Analyses of Gut Microbiota Composition and Diversity by 16S rRNA Sequencing

**Figure 3:**
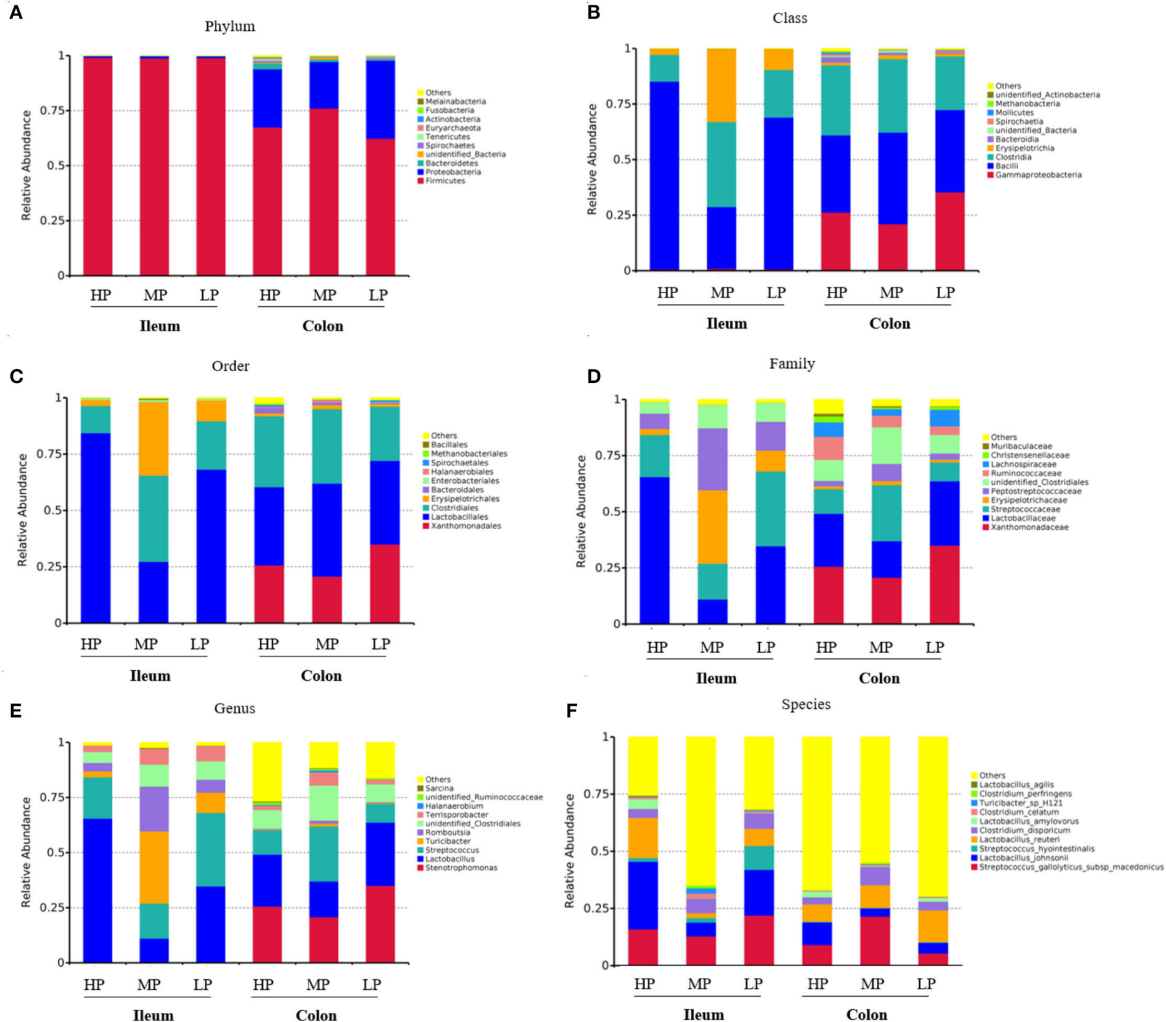
Effect of low-protein diet on the composition of gut microbiota at different taxa levels in finishing pigs. **(A)** Phylum, **(B)** class, **(C)** order, **(D)** family, **(E)** genus, and **(F)** species. HP, high protein (16% crude protein); MP, medium protein (12% crude protein); LP, low protein (10% crude protein).

As shown in Figure 3, the compositions of abundant bacteria (Top 10) are provided at phylum, class, order, family, genus, and species levels in the ileum and colon digesta of finishing pigs. At the phylum level, the dominant microbiotas were Firmicutes and Proteobacteria (Figure 3A). Firmicutes accounted for 99% of the ileum of finishing pigs from the three groups. The abundance of Firmicutes was 75% in the colon of finishing pigs of the MP group, 70% in the HP group, and 65% in the LP group (Figure 3A). Moreover, at the class level, the dominant microbiota in the ileum of the three groups were Bacilli, Clostridia and Erysipelotrichia, while in the colon is gamma Proteobacteria, Bacilli, and Clostridia (Figure 3B). Similar patterns were found at the order level (Figure 3C). At the family level, the Lactobacillaceae in the HP group of ileum was significantly higher than those of MP and LP groups (Figure 3D). In addition, the three dominant bacteria were *Stenotrophomonas, Lactobacillus*, and *Streptococcus* at the genus level (Figure 3E). At the species level, the dominant bacteria were *Streptococcus gallolyticus subsp macedonicu*s, *Lactobacillus johnsonii*, and *Streptococcus hyointestinalis* (Figure 3F).

**Figure 4:**
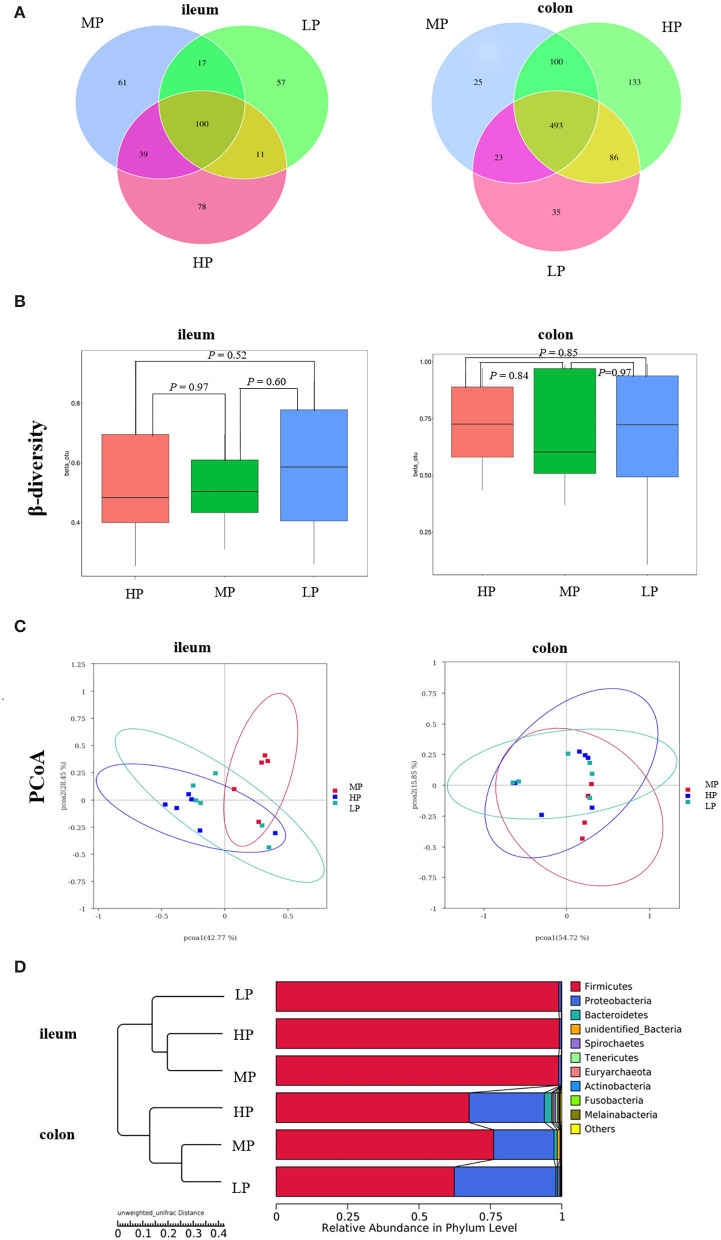
The Venn diagram and UPGMA cluster tree of gut microbiota in finishing pigs. **(A)** The Venn diagram. **(B)** β-diversity index. **(C)** PCoA plot. **(D)** UPGMA clustering was conducted based on unweighted unifrac distance. HP, high protein (16% crude protein); MP, medium protein (12% crude protein); LP, low protein (10% crude protein).

In the ileum, there were 100 common OTUs shared by the three groups, with 78, 61, and 57 unique to HP, MP, and LP, respectively (Figure 4A). In the colon, the number of common OTUs shared by the three groups was 493, and 133 (HP), 25 (MP), and 35 (LP) OTUs were unique to the treatments. Based on Bray-Curtis dissimilarity, the β-diversity index of ileal and colon microbiota was not significantly different among groups (*P* >0.05) (Figure 4B). However, the PCoA analysis revealed distinct differences in ileal microbiota between the MP group and HP group (Figure 4C). The **UPGMA** clustering tree based on unweighted UniFrac distance also demonstrated significant differences in ileal and colonic microbiota between the three treatments (Figure 4D). Moreover, the effect of the LP diet on the α-diversity of gut microbiota in finishing pig are summarized in Table 8. Especially, the observed species, Chao 1, PD_whole_tree of ileal microbiota were significantly decreased by both the HP and LP diets compared to the MP diets (*P* < 0.05). The LP diets also decreased the observed species of colonic microbiota in finishing pigs compared to the HP diet (*P* < 0.05).

**Table 8:** Effect of low protein diet on the α-diversity of gut microbiota in finishing pig.

**Item**	**HP**	**MP**	**LP**	***P*** **-value**
**Ileal digesta**				
Observed_species	69.00 ± 9.07^b^	121.33 ± 9.82^a^	73.40 ± 2.27^b^	0.002
Shannon	2.80 ± 0.22	3.04 ± 0.24	2.86 ± 0.30	0.808
Simpson	0.74 ± 0.04	0.77 ± 0.05	0.73 ± 0.08	0.932
Chao1	70.20 ± 10.65^b^	130.14 ± 18.52^a^	89.45 ± 3.25^b^	0.011
ACE	81.42 ± 12.47	101.08 ± 7.88	96.08 ± 3.62	0.345
Goods_coverage	1.00 ± 0.00	1.00 ± 0.00	1.00 ± 0.00	0.287
PD_whole_tree	4.97 ± 0.70^b^	10.49 ± 1.19^a^	5.24 ± 0.39^b^	0.001
**Colonic digesta**				
Observed_species	496.75 ± 38.53^a^	359.00 ± 37.96^b^	323.20 ± 14.48^b^	0.006
Shannon	4.95 ± 0.41	4.21 ± 0.41	4.13 ± 0.42	0.337
Simpson	0.87 ± 0.03	0.81 ± 0.05	0.80 ± 0.06	0.604
Chao1	512.56 ± 54.85	447.31 ± 46.37	389.01 ± 25.48	0.166
ACE	516.03 ± 54.42	435.82 ± 41.20	391.93 ± 15.82	0.122
Goods_coverage	1.00 ± 0.00	1.00 ± 0.00	1.00 ± 0.00	0.774
PD_whole_tree	36.58 ± 5.40	29.23 ± 1.48	24.11 ± 0.77	0.098

a, b *Within a row, means (n = 6) with a different superscript are significantly different at P < 0.05*.

*HP, high protein (16% crude protein); MP, medium protein (12% crude protein); LP, low protein (10% crude protein)*.

**Figure 5:**
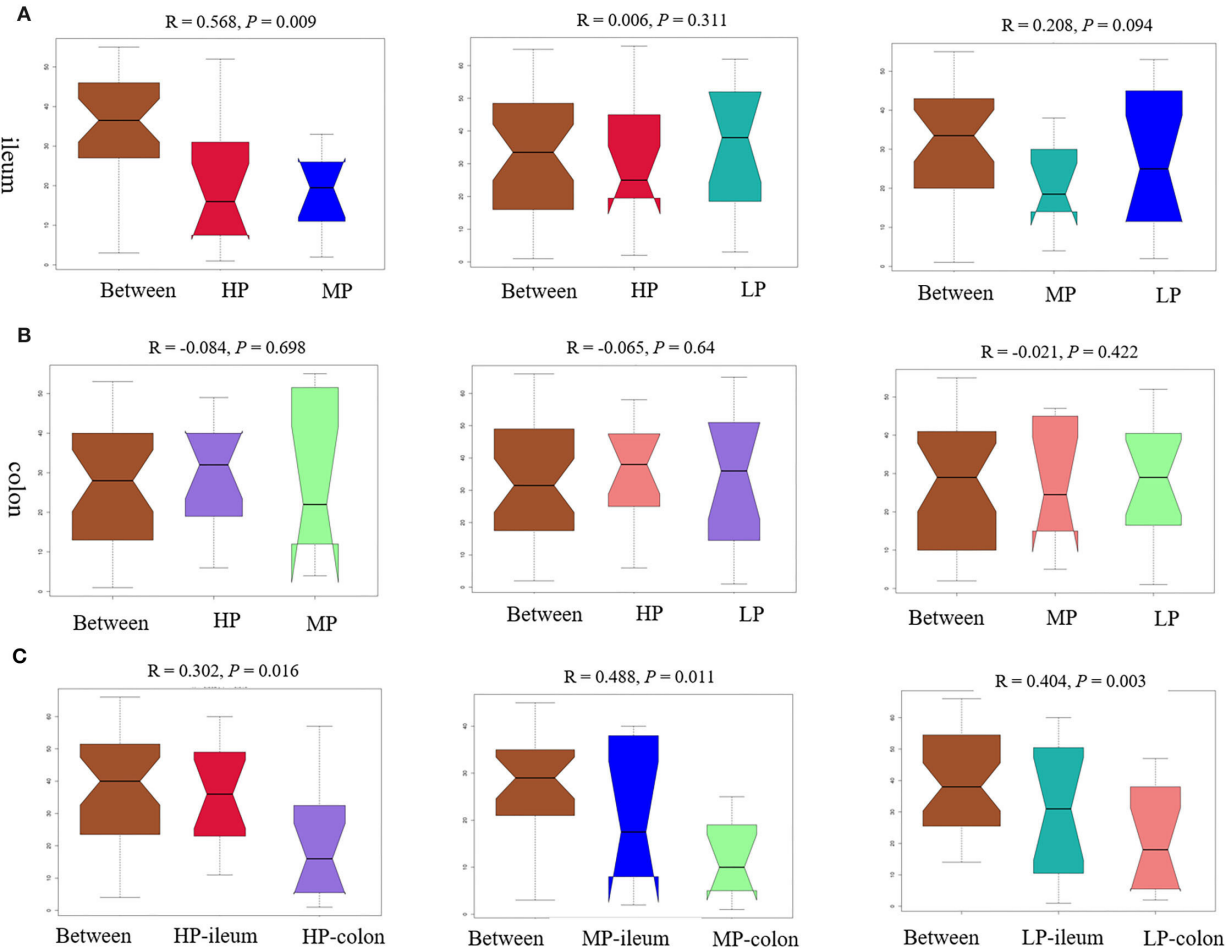
The analysis of similarities (ANOSIM) tests of gut microbiota. The ANOSIM tests were performed between groups based on relative abundance of OTU. ANOSIM results for ileal **(A)** and colonic **(B)** microbiota structure, as well as for the comparison between ileum and colon **(C)** from either HP, MP, or LP group. HP, high protein (16% crude protein); MP, medium protein (12% crude protein); LP, low protein (10% crude protein).

Furthermore, the ANOSIM (Figure 5A) showed that the structure of the ileal microbiota differed between MP and HP in finishing pigs (*R* = 0.568, *P* = 0.009). There was a change tendency in ileal microbiota structure between MP and LP (*R* = 0.208, *P* = 0.094), but no significant difference between HP and LP (*R* = 0.006, *P* = 0.311) in finishing pigs. However, there were no significant difference in colonic microbiota structure between HP and MP (*R* = −0.084, *P* = 0.698), as well as between HP and LP (*R* = −0.065, *P* = 0.64), or between MP and LP (*R* = −0.021, *P* = 0.422) in finishing pigs (Figure 5B). Notably, the microbiota structures between ileum and colon were significantly different from either HP (*R* = 0.302, *P* = 0.016), MP (*R* = 0.488, *P* = 0.011), or LP (*R* = 0.404, *P* = 0.003) group in finishing pigs, respectively (Figure 5C). Moreover, the Adonis results showed similar change patterns (Supplementary Table 1) as shown by ANOSIM.

**Figure 6:**
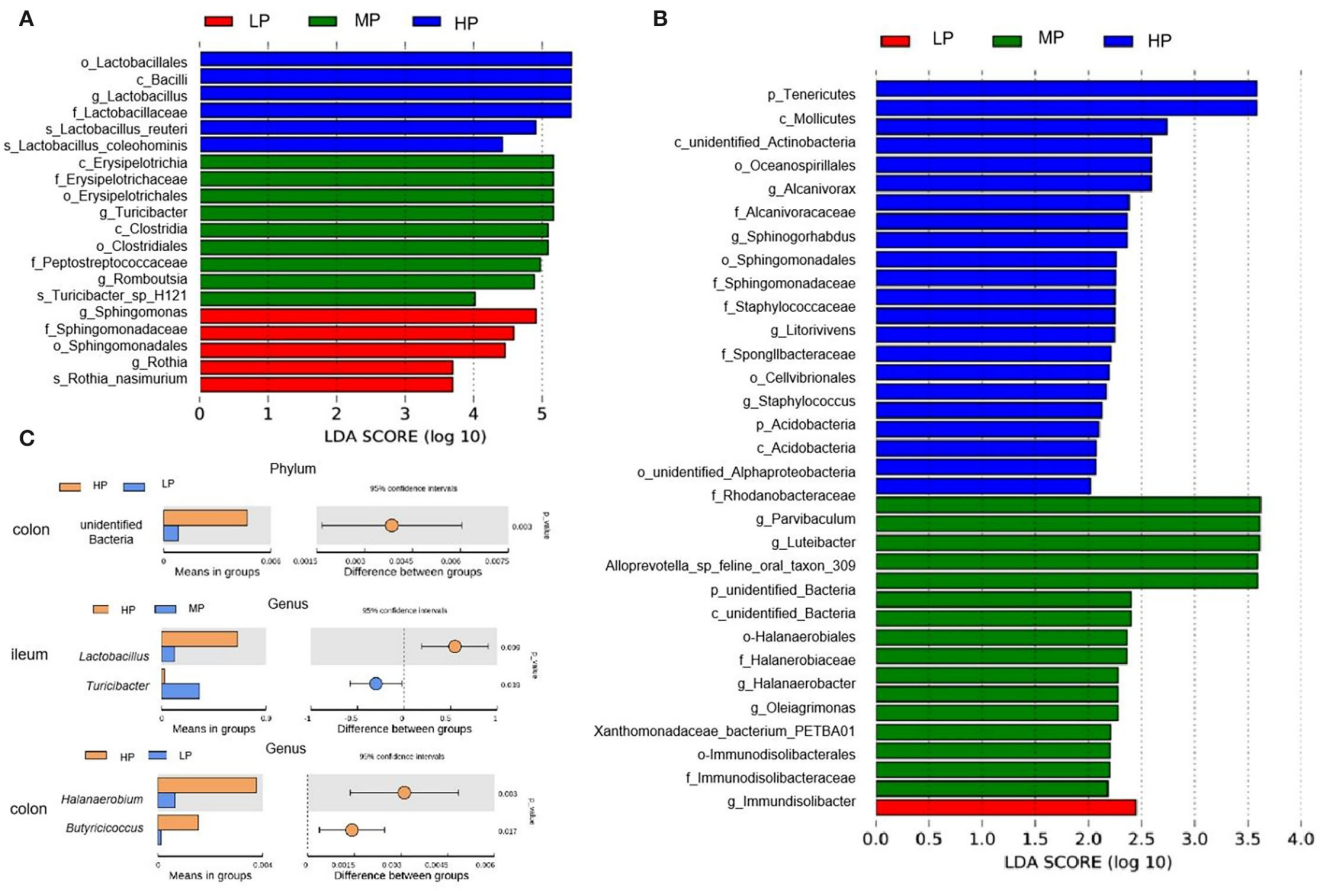
Linear discriminant analysis (LDA) effect size (LEfSe) and *t*-test analysis (*P* < 0.05, LDA > 2) of gut microbiota. LEfSe analysis for ileal **(A)** and colonic **(B)** digesta samples. **(C)** *T*-test analysis of gut microbiota. HP, high protein (16% crude protein); MP, medium protein (12% crude protein); LP, low protein (10% crude protein).

The LEfSe analysis showed the relative abundances of *Sphingomonas* and *Rothia* at the genus level, the Sphingomonadaceae at the family level, the Sphingomonadales at the order level, and the Rothia_nasimurium at the species level were significantly enriched in the ileum digesta of finishing pigs fed the LP diets (Figure 6A). Moreover, the LP diet also enriched the genus *Immundisolibacter* in the colonic digesta of finishing pigs (Figure 6B). Furthermore, the *t*-test analysis showed that the abundance of unidentified bacteria at the phylum level was significantly decreased by the LP diet when compared to the HP diet (Figure 6C). At the genus level, the LP diet significantly decreased the abundance of *Halanaerobium* and *Butyricicoccus* in the colonic digesta compared to the HP diet, while the HP diet increased the abundance of *Lactobacillus* compared to the MP diet in the ileal digesta (Figure 6C).

**Figure 7:**
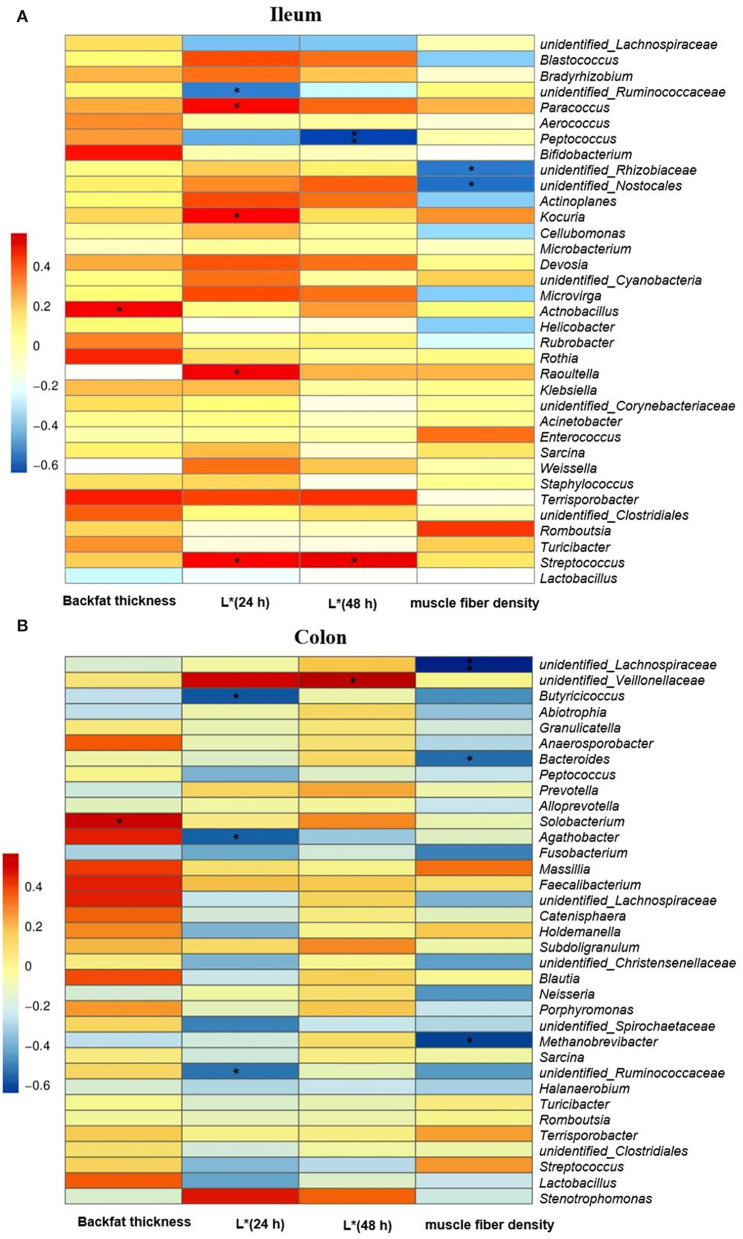
The Spearman correlation analysis of gut microbial composition at genus level with carcass trait and meat quality of finishing pigs. Spearman correlation coefficients of backfat thickness at first rib, meat color L* at 24 and 48 h and muscle fiber density with ileal **(A)** and colonic **(B)** microbiota, are represented by color ranging from red (positive correlation) to blue (negative correlation). * and **indicates statistically significant difference (*P* < 0.05) and (*P* < 0.01), respectively.

In the ileum (Figure 7A), the Spearman correlation analysis revealed that there was a positive correlation between the abundance of *Actnobacillus* at the genus level and the first rib backfat thickness in finishing pigs (*P* < 0.05). The relative abundances of the genera of *Paracoccus, Kocuria, Raoultella*, and *Streptococcus* were positively associated with meat color L^*^ value at 24 h, while that of unidentified_*Ruminococcasceae* was negatively correlated with L^*^ value at 24 h (*P* < 0.05). Moreover, there was a negative correlation between the abundance of *Peptococcus* at the genus level the and L^*^ value at 48 h (*P* < 0.01), and a positive correlation between the abundance of the genera *Streptococcus* and L^*^ value at 48 h (*P* < 0.05). Furthermore, the relative abundances of unidentified_*Rhizobiaceae* and unidentified_*Nostocales* at the genus level were negatively associated with muscle fiber density in finishing pigs (*P* < 0.05). In the colon (Figure 7B), there was a positive correlation between the abundance of *Solobacterium* at the genus level and backfat thickness. Moreover, a negative correlation was found between the abundance of the colonic genera of *Butyricicoccus, Agathobacter*, and unidentified_*Ruminococcasceae* and L^*^ value at 24 h (*P* < 0.05). In addition, the colonic unidentified_*Veilonellaceae* abundance was positively associated with L^*^ value at 48 h (*P* < 0.05). The muscle fiber density was negatively associated with the relative abundances of colonic unidentified_*Lachnospiraceae* (*P* < 0.01), *Bacteroides*, and *Methanobrevibacter* (*P* < 0.05).

## Discussion

Low protein (LP) diets are commonly used in growing-finishing pigs during swine production, especially for effectively reducing the discharge of nitrogen into the environment (13, 14), and improving carcass traits and meat quality (1, 15). However, whether the regulation of LP diet in regulating carcass composition and meat quality was associated with the modulation of gut microbiota in finishing pigs remains unclear. In this study, the effects of the LP diet supplemented with lysine, methionine, threonine, and tryptophan on growth performance, nitrogen emission, carcass traits, meat quality, antioxidant capacity, and gut microbiota in finishing pigs were investigated. The present results showed that there was no significant difference in ADG, ADFI, and F/G among the three diets, which suggested that LP diets with balanced AAs and energy had no negative effect on the performance of finishing pigs over 100 kg. Our results were consistent with previous studies of LP diets conducted in weaned piglets (16, 17), growing and finishing pigs (18–20), chickens (21), and lambs (22). For example, the reduction of dietary CP level from 17 to 15% did not impair the growth performance in pigs of 15–35 kg but reducing dietary CP level to 13% would lead to the decrease of BW, ADG, and ADFI and the increase of F/G during this phase (18). Moreover, reducing dietary PC levels from 14.5 to 10% had no negative effect on the growth performance in finishing pigs over 75 kg (19). Lowering dietary CP by 3% units with lysine supplementation showed similar growth performance of growing-finishing pigs of 35–180 kg to those fed the NRC (2012) recommended level (17).

Dietary high CP level would increase the excretion of excessive nitrogen, reduce the efficiency of nitrogen utilization, and damage gut health by more protein fermentation in the hindgut (1). Many studies have shown that the reduction of dietary CP levels could effectively reduce the fecal and/or urinary nitrogen excretion in growing pigs (23), finishing pigs (13), primiparous gilts (24), as well as growing goats (25), and decrease blood urea nitrogen concentration in weaned piglets (26) and mice (27). Moreover, lowering dietary CP levels significantly reduced nitrogen consumption without negative consequences on growth performance in growing bulls (28). We also found that LP diets induced a significant decrease in the plasma urea nitrogen contents and fecal nitrogen excretion in finishing pigs of 100–130 kg. The reduction of nitrogen emission might be associated with the greater abundance of urease-producing bacterial species detected in LP diets (27). Therefore, the results from our study and others suggest that the application of the LP diet represents an effective environmental-friendly way to reduce the feed cost and nitrogen emission without affecting the performance of finishing pigs.

Carcass composition traits and meat quality are becoming important in both animal breeding and nutrition regulation due to their high economic value. Previous studies have shown that decreasing dietary CP levels would not affect carcass traits in growing bulls (28), or the carcass yield in Charolais bulls (29). In this study, most of the carcass traits were not significantly affected by dietary protein levels, and no significant differences were found in carcass weight of finishing pigs when fed with LP diets. Consistently, similar results in Iberian pigs also demonstrated that the carcass weight and carcass yield did not differ in response to the LP diets (30). However, the backfat thickness was significantly increased by the LP diet in the present study, which was consistent with previous results that LP diets induced higher backfat thickness in pigs at 30, 60, and 100 kg (31). The increased backfat thickness of LP diets might be due to the excess energy deposited in the form of body fat, but further investigations are needed to elucidate the potential mechanism.

Importantly, the protein levels in commercial diets have a critical impact on pork meat quality (6). Meat color is the most important appearance quality trait by the consumer and is used as an indicator of freshness and wholesomeness (32). In this study, we found that the LP diet improved the meat quality by improving the meat color in finishing pigs, especially for the yellowness (b^*^ value) of 24 and 48 h. Similarly, a recent study also showed that the LP diet was associated with changes in meat color intensity, drip loss, and muscle fiber area in chickens (33). Indeed, dietary protein intake has been shown to regulate the expression of key genes for lipid metabolism in the skeletal muscle of finishing pigs (34). The LP diet improved meat quality by changing lipid metabolism, fiber characteristics, and the free AA profile of pig muscle (35). To explain the potential mechanism involved, the expression of genes related to fat metabolism and muscle fiber was also determined in this study. Our results showed that the LP diets significantly downregulated the expression of FAS, PPAR-γ, leptin, and ACC, but upregulated the expression of CPT1 and MyHC IIx in the skeletal muscle of finishing pigs. Our results were consistent with the previous study that the LP group significantly increased the^*^ value due to the upregulated expression of MyHC I and MyHC IIa in both growing pigs and finishing pigs (13). A previous study also suggested that the improvements in meat quality in finishing pigs by LP diets could be partially related to the regulation of ACCα and HSL expression (36, 37). In contrast, the fat deposition in Wujin pigs fed HP diets for 25 weeks was reduced mainly by depression of lipogenic gene expression in porcine adipose tissue (31). Besides, the antioxidant capacity has been indicated to be associated with meat quality traits (20). A previous study showed that a dietary lower methionine and cysteine ratio in an LP diet improved antioxidant capacity in boar (38), while the extreme LP diet might not support well the antioxidant defense and ability to combat the stress in *Chrysolophus amherstiae* (39). Here, we also found that the LP diets reduced plasma MDA contents and increased the GSH contents in the plasma of finishing pigs. Collectively, the LP diets could increase meat color and muscle fibers density, which might partially involve the modulation of the gene expression related to fat metabolism and antioxidant capacity.

Increasing evidence has demonstrated the interaction between gut microbiota and host health and disease in domestic pigs (40–42). Notably, dietary protein composition and source could modulate the composition, activity, and function of gut microbiota (42, 43). Importantly, the LP diets with balanced AA display beneficial effects for maintaining gut health and improving the digestion and absorption function by modulating the composition and diversity of gut microbiota as well as the functions of microbial metabolism (44–47). For example, the bacterial community, especially for the short-chain fatty acid (SCFA) producing bacteria was increased while *Escherich coli* numbers and the protein fermentation products were decreased by LP diets (48). Moreover, the relative abundance of *Lactobacilli* in the cecum and that of *Streptococcus* in the colon of growing pigs was decreased when fed diets reduced by 3% CP compared with an HP diet (49). Similar results were found in broilers that reducing CP intake increased the relative abundance of Lactobacillaceae in the cecum at the family level probably linking to a better FCR (21). By these results, we also found that the LP diet induced a significant decrease of *Lactobacillus* abundance in the ileal digesta of finishing pigs compared to the HP diet in the present study. Similarly, a recent study also showed that the LP diet increased intestinal microbiota species and richness indices in both barrows and gilts, and the relative abundances of unidentified *Clostridiales, Neisseria*, unidentified *Prevotellaceae*, and *Gracilibacteria* at genus level were significantly affected by dietary CP levels (50). Consistently, we found that the LP diet significantly affected the diversity index of microbiota in the ileal digesta of finishing pigs. Furthermore, the LP diet significantly decreased the abundance of unidentified Bacteria at the phylum level, and *Halanaerobium* and *Butyricicoccus* at the genus level in the colonic digesta. Notably, gut microbiota possibly exerts essential roles in controlling nitrogen utilization of gilts fed LP diets with fiber supplementation (51). Thus, the composition and diversity of gut microbiota in finishing pigs were affected by dietary CP level, and LP diets could modulate the microbial community and diversity, which might be associated with changes in carcass traits and meat quality. In recent years, the gut microbiota has been recognized as an important factor contributing to the regulation of skeletal muscle mass and function (51) as well the fat deposition (52). Interestingly, targeting gut microbiota facilitates the dietary protein efficacy to prevent the diminished skeletal muscle mass and strength during aging (53). The Spearman correlation analysis confirmed that regulation of gut microbiota composition by LP diets correlated with changes in backfat thickness, meat color, and muscle density in this study. This was consistent with a previous study that gut microbiota had the potential to improve the meat quality and flavor of pigs by regulating the lipid metabolism of porcine skeletal muscle (54). However, further studies concerning the link of the “microbiota-muscle axis” and the regulative mechanism of key gut microbiota and its metabolites on pork quality during future implications of LP diets in pigs are necessary.

In conclusion, the present results indicate that the LP diet supplemented with lysine, methionine, threonine, and tryptophan had no detrimental impacts on the growth performance of finishing pigs over 100 kg, but significantly improved the meat quality by modulating the muscular gene expression involved in fat metabolism, and antioxidant capacity as well as the composition of gut microbiota in finishing pigs.

## Data Availability Statement

The original contributions presented in the study are publicly available. This data can be found here: Submission ID: SUB11431863, BioProject ID: PRJNA835299. The direct link/URL to this data is https://www.ncbi.nlm.nih.gov/bioproject/PRJNA835299/.

## Ethics Statement

The animal procedures used in this study were approved by the Animal Care and Use Committee of Guangdong Academy of Agricultural Sciences (Guangzhou, China).

## Author Contributions

CZ, LW, and ZJ: conceptualization and writing-reviewing. CZ and JY: data analyses and writing-original draft preparation. JY, QW, JC, and XY: sample collection and analyses. CZ and ZJ: funding acquisition. All authors contributed to the article and approved the submitted version.

## Funding

The authors gratefully acknowledge the financial support from the National Key Research and Development Program of China (2021YFD1300004), the Open Project of State Key Laboratory of Livestock and Poultry Breeding (2021GZ03), the China Agriculture Research System of MOF and MARA, the Guangdong Basic and Applied Basic Research Foundation, China (2020A1515010018 and 2022A1515011185), the Science and Technology Program of Guangdong Academy of Agricultural Sciences (R2020PY-JG009), and the Independent Research and Development Projects of Maoming Laboratory (2021ZZ003).

## Conflict of Interest

The authors declare that the research was conducted in the absence of any commercial or financial relationships that could be construed as a potential conflict of interest.

## Publisher's Note

All claims expressed in this article are solely those of the authors and do not necessarily represent those of their affiliated organizations, or those of the publisher, the editors and the reviewers. Any product that may be evaluated in this article, or claim that may be made by its manufacturer, is not guaranteed or endorsed by the publisher.
