# Supplementary material for: Low Protein Diet Improves Meat Quality and Modulates the Composition of Gut Microbiota in Finishing Pigs
Source: Front Vet Sci. 2022 May 17;9:843957. doi: 10.3389/fvets.2022.843957 (PMC9152361; doi:10.3389/fvets.2022.843957)
Supplement: Supplementary file 1 [file Table_1.DOCX]

**Table S1**. The Adonis test of gut microbiota in finishing pigs

| **Adonis** | ***P* value** |
| --- | --- |
| **Ileum** |  |
| LP vs. MP | 0.078 |
| LP vs. HP | 0.351 |
| MP vs. HP | 0.004 |
| **Colon** |  |
| LP vs. MP | 0.323 |
| LP vs. HP | 0.644 |
| MP vs. HP | 0.588 |
| **Ileum vs. Colon** |  |
| HP ileum vs. HP colon | 0.007 |
| MP ileum vs.colon | 0.014 |
| LP ileum vs.colon | 0.002 |

Abbreviations: HP = high protein (16% crude protein), MP = medium protein (12% crude protein), LP = low protein (10% crude protein).
